# Measuring grief and loss after spinal cord injury: Development, validation and psychometric characteristics of the SCI-QOL Grief and Loss item bank and short form

**DOI:** 10.1179/2045772315Y.0000000015

**Published:** 2015-05

**Authors:** Claire Z. Kalpakjian, David S. Tulsky, Pamela A. Kisala, Charles H. Bombardier

**Affiliations:** 1 Department of Physical Medicine and Rehabilitation, University of Michigan Medical School, University of Michigan, Ann Arbor, MI, USA; 2 New York University Langone Medical Center, New York, NY, USA; 3 Kessler Foundation Research Center, West Orange, NJ, USA; 4 Department of Rehabilitation Medicine, University of Washington, Seattle, WA, USA

**Keywords:** Grief, Spinal cord injuries, Patient outcomes assessment, Quality of life, Psychometrics

## Abstract

**Objective:**

To develop an item response theory (IRT) calibrated Grief and Loss item bank as part of the Spinal Cord Injury – Quality of Life (SCI-QOL) measurement system.

**Design:**

A literature review guided framework development of grief/loss. New items were created from focus groups. Items were revised based on expert review and patient feedback and were then field tested. Analyses included confirmatory factor analysis (CFA), graded response IRT modeling and evaluation of differential item functioning (DIF).

**Setting:**

We tested a 20-item pool at several rehabilitation centers across the United States, including the University of Michigan, Kessler Foundation, Rehabilitation Institute of Chicago, the University of Washington, Craig Hospital and the James J. Peters/Bronx Department of Veterans Affairs hospital.

**Participants:**

A total of 717 individuals with SCI answered the grief and loss questions.

**Results:**

The final calibrated item bank resulted in 17 retained items. A unidimensional model was observed (CFI = 0.976; RMSEA = 0.078) and measurement precision was good (theta range between −1.48 to 2.48). Ten items were flagged for DIF, however, after examination of effect sizes found this to be negligible with little practical impact on score estimates.

**Conclusions:**

This study indicates that the SCI-QOL Grief and Loss item bank represents a psychometrically robust measurement tool. Short form items are also suggested and computer adaptive tests are available.

## Introduction

Loss is a universal experience. Bereavement, the loss of a loved one, and grief, the distress resulting from bereavement,^1^ are concepts that patients and their loved ones naturally use to understand reactions to catastrophic losses like spinal cord injury (SCI). However, questions remain about the applicability of grief and bereavement to understanding adjustment to SCI.^2^ Most salient is that grief and bereavement classically focus on the loss of a loved one. Grief also has been used to characterize reactions to other losses, but the extent to which models of grief can be applied to SCI-related loss of physical capacity, social or occupational role function, and life goals remains an empirical question. Moreover, measuring grief and loss, the focus of this paper, must be distinguished from theories about the *process* of grief, especially those characterized by Kubler-Ross' stages of grief.^3^

Despite its heuristic appeal, grief is an abandoned concept in SCI rehabilitation. Without empirical support, grief was thought to be universal after injury, required to achieve positive adjustment, and occurring in a particular way, commonly described as ‘stage theory.’^4,5^ Grief also lacked a strong foundation in measurement. Rehabilitation psychologists eventually moved towards the measurement of core psychopathological constructs, such as depression^6^ and post-traumatic stress disorder,^7^ as well as positive psychological constructs, such as coping and appraisal.^8,9^

In the meantime, significant theoretical, empirical, measurement, and treatment advancements have occurred in the field of grief and bereavement. In terms of theory, Stroebe and Shut^10^ have described the influential dual-process model of coping with bereavement. From this perspective, the bereaved are thought to oscillate between a focus on loss and a focus on restoration in daily activities. In a groundbreaking *prospective* study of spousal bereavement, Bonanno *et al.*^11^ described five trajectories of responses to loss, including resilience as the modal response. Since then, similar trajectories have been demonstrated in response to other forms of trauma, including SCI.^12,13^ Newer conceptualizations of grief also suggest that the *context of the loss*, characterized by factors such as suddenness, unexpectedness, or violence, may be critical predictors of outcome.^14^

Controversy remains about how to define concepts such as normal, complicated and prolonged grief. Nevertheless, there is growing evidence that pathological grief occurs in a significant minority of people who sustain loss and is distinct from ‘normal’ grief, anxiety, and depression.^15^ Furthermore, pathological grief is associated with functional impairment, physiological changes, reduced quality of life, poor self-care, and elevated suicidal ideation and suicide attempts, after controlling for depression and anxiety.^15^ Prigerson *et al.*^15^ posit that key symptoms of pathological grief include yearning for what was, accompanied by other cognitive, emotional and behavioral symptoms, such as difficulty accepting the loss, emotional numbness, avoidance of reminders and confusion about one's role in life.

Distinguishing abnormal grief from depression also may have significant treatment implications. An influential clinical trial based on the dual process model of grief demonstrated that interpersonal therapy (IPT) alone was not as effective a treatment for complicated grief as was a combination of IPT and exposure therapy.^16^ *Making meaning of losses* is another emerging dimension of grief and bereavement, particularly when the loss is sudden or unexpected.^17^ The search for meaning after injury has been associated with positive adaptation to injury.^18^ Qualitative work suggests a process of adaption and meaning-making, characterized by seeking a balance between holding onto pre-injury goals and adaptation to life after injury.^19^

Given theoretical advances in the field of grief and bereavement, we speculated that these perspectives may also benefit our understanding of adjustment to losses associated with SCI. The concept of examining trajectories of adjustment has already been applied to people with SCI; however, with few exceptions,^20^ assessment of adjustment to SCI has been limited to measures of depression and anxiety.^13,21^ Popular scales such as Core Bereavement Items,^22^ Texas Revised Inventory of Grief,^23^ or the Inventory of Complicated Grief^24^ focus on the loss of a loved one. Construct validity of such scales is examined almost exclusively in respondents who have experienced the death of a loved one. As such, the usefulness and validity of such items is limited for persons with SCI, where losses are focused not on the loss of another, but on losses related to the self. To address the gap in the availability of relevant and valid measurement tools to measure grief processes after SCI, we sought to create a new item bank to capture features of grief/loss that were highly relevant to the losses experienced by persons with SCI.

## Methods

This study was approved by the Institutional Review Board at all sites. The first study activity was to develop and refine a Grief and Loss item pool. Next, grief and loss items were administered to a large sample of people with SCI using a computerized data collection platform and interview format, so that each question was read to the respondent by a trained interviewer and responses were directly entered into the database. Each of these steps is described in detail in Tulsky *et al.*^25^ and is also outlined briefly in the section below.

### Development of a grief and loss item pool

The Grief and Loss item bank assesses emotional reactions or grief such as anger, guilt, anxiety, sadness, and despair. Items comprising the Grief and Loss item bank were drawn from various sources. We began by identifying candidate items from our initial pilot work, which included individual, semi-structured interviews and focus groups with individuals with SCI and clinicians with SCI (see Tulsky *et al*.^25,^^26^ for a full description). From these data, we developed a set of two preliminary items related to grief and loss. To develop these new items, specific phrases or concepts were drawn from the interviews and focus group transcripts (12 items from pilot interviews and 20 items from focus groups). For example, from the patient focus group quote, ‘*[T]he first thing that came to my mind is, I guess, the things that you miss, you know, that you could do before. Yeah, even people say you can do them, you can do them in a different way, but I can't walk my dog effortlessly like I used to. If he gets loose, I can't just go over there and grab him and bring him back…*’ we drafted the item, *It made me sad to think about the things I used to enjoy*. Additionally, one item from the Neuro-QOL^27^ Stigma bank, *I lost friends by telling them that I have this [injury]*, was originally categorized as a SCI-QOL Grief/Loss item raising the total pool to 33 items.

Expert item review (EIR), the first phase of the qualitative item review process used by the PROMIS^28^ and Neuro-QOL^27^ project teams, was used to optimize this preliminary pool of 33 items. Items were considered candidates for deletion if they were redundant, overly generic, too specific, or if they failed to represent the construct or domain definition. Items that were verbose, double-barreled (i.e. one item with content related to two different issues), poorly or inconsistently worded, or contained high-level vocabulary were revised. For example, the item ‘It made me sad to think about the things I used to enjoy’ (mentioned above) was to read ‘I missed the activities I used to do.’ Based on EIR feedback, 30 items were retained in the preliminary Grief and Loss item pool. Preliminary items then underwent an additional phase of item review and modification by members of the investigative team. Items were arranged on a hierarchy of ‘difficulty’, from items indicating the lowest degree of grief and loss to the highest degree of grief and loss. Team members removed redundant items where there was oversaturation in the middle range of the hierarchy, and suggested new items to fill gaps in content coverage. Specifically, 3 items were deleted during this phase of review, 6 were moved to other item banks (e.g. Trauma, Stigma) and 5 new items were added.

We then conducted cognitive interviews^29^ with at least five individuals with SCI (see Introduction paper in this issue for details on the methodology) to assess item comprehension, decision making, and response retrieval processes. After cognitive interviewing, 7 items were removed and 4 items were modified. After this phase, the final 20 items were reviewed for translatability (for method, please see Eremenco *et al*.^30^) and reading level (using the Lexile framework^31^). Slight modifications were made to 3 items after the translatability and cultural review. For example, the item ‘I was upset about everything that has happened to me’ was changed to ‘I was overwhelmed by everything that has happened to me,’ since there is no generic word for ‘upset’ in some other languages such as Spanish. All items were written at or below the 5th grade reading level. A final item pool of 20 items was then field-tested.

### Calibration study participants and data collection procedures

As a part of a large-scale, multi-site item calibration study (sites included the Kessler Foundation, University of Michigan, Rehabilitation Institute of Chicago, University of Washington, Craig Hospital and the James J. Peters/Bronx Veterans Administration hospital), we administered the 20 grief and loss items along with other item pools reflecting different emotional health subdomains to a sample of people with SCI.

The calibration sample included 717 participants with SCI. Inclusion criteria were 18 years of age and older, ability to read and understand English, and had a medically documented traumatic SCI. The sample was stratified by level (paraplegia versus tetraplegia), completeness of injury (complete vs. incomplete), and time since injury (<1 year, 1–3 years, and >3 years) to ensure that the final sample was a heterogeneous sample of individuals with SCI. Each participant's diagnosis was confirmed by medical record review; neurologic level was documented by their most recent American Spinal Injury Association Impairment Scale (AIS) rating.^32^ To ensure a consistent mode of administration across participants, all items were presented in a structured interview format, either in person or over the phone. The context (time frame) for all items was, ‘In the past 7 days…,’ and the response options were Never/Rarely/Sometimes/Often/Always. A more detailed description of the study methodology and procedures is provided elsewhere in this issue.^25^

### Data analyses

Analysis involved confirmation of construct unidimensionality, use of a graded-response IRT model to calibrate item parameters, and examination of DIF. We used CFA to determine if our items conformed to a unidimensional model. Acceptable model fit indices were: CFI > 0.90, RMSEA < 0.08, good; CFI > 0.95, RMSEA < 0.06, excellent). Calibration was performed using iterative methods to reduce the item pool and obtain the best-fitting item parameters that would best allow estimation of a participant's standing on a trait of grief and loss. With each successive analytic iteration, we identified poorly fitting items by examining item fit to the 2-PL IRT model, DIF, local dependence between items (residual correlations >|0.15|), and significant loadings on the single factor (values >0.30). We then removed these items from the item pool and repeated the analytic steps. Once an acceptable solution was reached with CFA statistics that supported a unidimensional model, and all items showing misfit to the model or DIF were removed, the final IRT parameters were utilized to develop a computer adaptive test (CAT) algorithm for the Grief and Loss item bank. The CAT was programmed on the Assessment Center website (http://www.assessmentcenter.net) and can be administered directly from there. The item parameters were also used to select items for a static short form which can also be downloaded as a PDF from the Assessment Center website. Tulsky *et al*.^25^ within this special issue described the detailed methodology and data analysis plan.

### Development of short forms

To select items for short, fixed-length forms (as an alternative to CATs), project investigators reviewed item difficulty (item location) and slope (discrimination). As a starting point, items were divided into quintiles based on location; at each quintile, the first and/or second items with the highest slope were selected. Other considerations for item selection were clinical relevance, item wording and similarity to other items with the goal of having short form items as diverse as possible. Therefore, selection of items for short forms used both item statistics and qualitative characteristics.

## Results

### Participant characteristics

Demographic and injury characteristics are summarized in Table 1. Please see Tulsky *et al*.^33^ introductory article within this special issue for additional details on the calibration sample, including education, income and mechanism of injury.

**Table 1:** Demographic and injury characteristics of the calibration sample

Variable	Emotional domain sample, *N* = 716; Mean (SD), *N* (%)
Age (years)	43.0 (15.3)
Age at injury (years)	36.1 (16.8)
Sex	
Male	558 (78%)
Female	158 (22%)
Ethnicity	
Hispanic	81 (11%)
Non-Hispanic	631 (88%)
Not reported/Refused	4 (1%)
Race	
Caucasian	505 (70%)
African-American	125 (17%)
Asian	8 (1%)
American Indian/Alaska Native or Native Hawaiian/Pacific Islander	7 (1%)
More than one race	9 (1%)
Other	49 (7%)
Not provided/Refused	4 (1%)
Level of Education	
High school or less	274 (38.3%)
Some college	248 (34.6%)
Bachelor's degree or more	194 (27.1%)
Time Since Injury	7.1 (10.0)
<1 year post injury	195 (27%)
1–3 years post injury	186 (26%)
>3 years post injury	335 (47%)
Diagnosis	
Paraplegia complete	182 (25%)
Paraplegia incomplete	143 (20%)
Tetraplegia complete	157 (22%)
Tetraplegia incomplete	230 (32%)
Unknown	4 (0%)

### Preliminary analysis and item removal

Of the original 20 items that were tested, 3 were removed for the following reasons: local item dependence, low item-total correlation, and DIF for gender (Grief_31, “I cried when I was reminded of the abilities I used to have”), respectively. For the final 17 retained items, internal consistency was excellent (Cronbach's *α* = 0.947) and item-total correlations ranged from 0.59 to 0.78. All of the items but one had more than 20% of the sample selecting the first category of category of 1 (‘Never’). One case was deleted due to excessive missing data. No items had sparse data (fewer than five responses) in any category and no items had a category inversion. No further items were removed at this time.

### Dimensionality

Using CFA, a unidimensional model was observed (CFI = 0.976; RMSEA = 0.078). The *R*^2^ values for all 17 of the items were greater than 0.40 In terms of local dependence, no item pairs were identified (i.e. residual correlations >|0.15|). Eigenvalue ratio (first to second) was 11.8.

### IRT parameter estimation and model fit

Slopes ranged from 1.65 to 3.15; thresholds ranged from −1.48 to 2.48 (see Table 2).

**Table 2:** Grief and Loss items and item bank parameters

		Item response theory calibration statistics				
Item ID	Item stem	Slope	Threshold 1	Threshold 2	Threshold 3	Threshold 4
**Grief_14**	**I spent a lot of time thinking about what I have lost since my injury**	**2.08941**	**−0.79108**	**0.07601**	**0.91063**	**1.73104**
Grief_16	I felt sad thinking about things I used to enjoy	3.00519	−0.71947	−0.11166	0.78169	1.48040
Grief_15	Because of my injury, I felt like I lost many opportunities	2.49928	−0.75116	−0.22986	0.56348	1.21498
**Grief_29**	**I felt that I lost my former life**	**3.01344**	**−0.44494**	**0.02941**	**0.70042**	**1.21062**
**Grief_10**	**I had difficulty accepting my injury**	**2.18233**	**0.05849**	**0.64106**	**1.37461**	**1.82508**
**Grief_7**	**I longed for the life I had before my injury**	**2.61178**	**−0.94542**	**−0.33790**	**0.41467**	**0.91664**
Grief_2	I missed out on life because of my injury	2.53096	−0.45129	0.10510	0.88385	1.51899
Grief_21	I have lost spontaneity in my life.	1.64954	−0.45633	0.21509	1.13592	2.04732
**Grief_13**	**Because of my injury, I was distressed about the abilities that I have lost**	**2.91124**	**−0.72949**	**−0.08964**	**0.81757**	**1.59383**
Grief_30	I was overwhelmed by everything that has happened to me	1.74946	−0.00510	0.80341	1.82045	2.47958
**Grief_28**	**I missed the activities I used to do**	**2.44946**	**−1.48060**	**−0.93461**	**0.04140**	**0.68014**
Grief_20	Because of my injury, I had difficulty adjusting to the changes in my body	1.84725	−0.40213	0.25354	1.46142	2.35339
**Grief_11**	**I felt that my injury has taken away my future**	**3.15448**	**−0.19293**	**0.22748**	**1.06119**	**1.54145**
Grief_9	I questioned why I was injured	1.69676	0.16936	0.69776	1.49720	2.00782
**Grief_6**	**I felt lost because of my injury**	**2.92100**	**0.30080**	**0.78731**	**1.62381**	**2.35060**
Grief_1	I felt I lost time because of my injury	2.10622	−0.62952	−0.14542	0.70884	1.45129
**Grief_24**	**I felt that I am not who I used to be**	**2.37777**	**−0.67015**	**−0.06706**	**0.68927**	**1.25193**

Context for all grief and loss items was ‘In the past 7 days…’; Response set was 1 = Never/2 = Rarely/3 = Sometimes/4 = Often/5 = Always. **Items in bold** represent short form selections. Items and parameters copyright © 2015 David Tulsky and Kessler Foundation. All Rights Reserved. Scales should be accessed and used through the corresponding author or http://www.assessmentcenter.net. Do not modify items without permission from the copyright holder.

**Figure 1:**
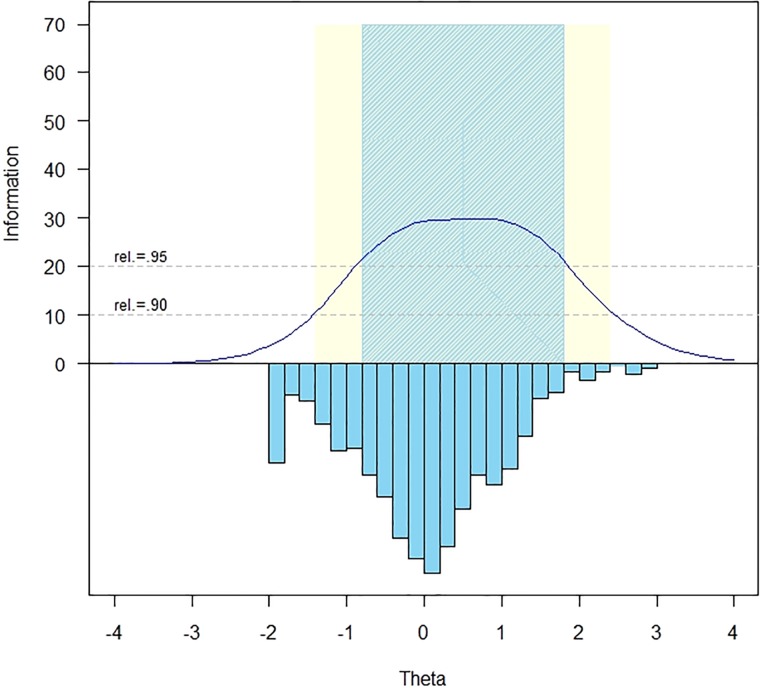
SCI-QOL Grief and Loss Item Bank Information and Precision.

The measurement precision in the theta range between −0.8 and 1.8 is roughly equivalent to a classical reliability of 0.95 or better (Fig. 1).

The S-X^2^ model fit statistics were examined using the IRTFIT macro program. All items had adequate or better model fit statistics (P < 0.05), with marginal reliability equal to 0.947 and no item pairs were flagged (|*r*|> = 0.4) for local dependence.

### Differential item functioning

DIF was examined using lordif^34^ for six categories: age (≤49 vs. ≥50), sex (male *n* = 559 vs. female *n* = 158), education (some college and lower *n* = 523 vs. college degree and above *n* = 194), diagnosis (tetraplegia *n* = 388 vs. paraplegia *n* = 325), injury severity (incomplete *n* = 374 vs. complete *n* = 339), and time post injury (<1 year *n* = 196 vs. >1 year *n* = 521). Items were flagged for possible DIF when the probability associated with the *χ*^2^ test was <0.01 and the effect size measures (McFadden's pseudo *R*^2^) >0.02, which is a small but non-negligible effect. Overall, 10 of the final items were flagged for DIF in at least one category based on the chi-square test; however, when the effect size measures were examined, the DIF was negligible and all 17 items were retained in the final, calibrated item bank.

### Short form selection and mode of administration

Once the SCI-QOL Grief and Loss item bank was finalized, all items and parameters were programmed into the Assessment Center^SM  35^ platform and the bank is now freely available as a CAT. Since the purpose of calibrating items using IRT is that only a subset of items needs to be administered from a given bank in order to estimate an individual's score, there is flexibility as to how the items are selected and administered. On the Assessment Center platform, the CAT administration parameters can be modified to reduce standard error variance (e.g. maximize reliability), or to reduce test burden. There is also a predetermined static short form that can be downloaded. Finally, the individual items are present and could be selected if the end user wanted to administer a specific item. These administration options are reviewed below.

The SCI-QOL utilizes the same default CAT discontinue criteria as PROMIS; namely, the CAT minimum number of items to administer is four and the maximum is 12 with a maximum standard error of 0.3. In other words, in the default settings, the CAT will always administer at least 4 items, then will discontinue when the standard error of the individual's score estimate drops below 0.3 or a maximum of 12 items is reached (and the standard error variance criterion cannot be met).

Alternatively, the user could change the ‘discontinue criteria’ of the CAT so that it will administer additional items and obtain a more precise assessment of functioning. For instance, if the user selected an option that the CAT administers a minimum of 8 items before discontinuing, a lengthier test would be administered, but a more reliable score will be obtained. In some cases, greater precision over test burden is desirable based on factors such as resource allocation where specificity is critical.

However, in some cases it is neither possible (e.g. internet unavailable) nor practical (e.g. laptop/tablet computer equipment beyond budget of project) to administer items via CAT. To address this need, the Grief and Loss and other SCI-QOL item banks are also available as short forms. The project investigators utilized psychometric and clinical input to develop a fixed, 9-item ‘short form’ version of the Grief and Loss item bank. The goal of the short form selection process was to include the most informative items across a wide range of ‘difficulty’, or amount of the underlying trait. Since all items are calibrated on the same metric, scores on the short form are directly comparable to those on the CAT or full item bank. The correlation of the short form and various CATs with the full bank are given in Table 3. Short forms may be administered directly within Assessment Center, or may be downloaded for administration by paper and pencil or an alternate data capture platform or system. Individual investigators or clinicians could also develop additional, custom short forms, which could then be scored on the same IRT-based metric with the help of a psychometrician.

**Table 3:** Accuracy of variable- and fixed-length CAT and 9-item short form: correlations with full-bank score

Mode	*N*	# Items admin				%Min	%Max	Corr. w/Full bank
		Mean	SD	Min	Max			
Variable-length CAT (min 4)	716	6.0	2.7	4	12	42.7	13.0	0.98
Variable-length CAT (min 8)	716	8.6	1.4	8	12	82.3	13.0	0.99
9-Item fixed-length CAT	716	9	0	9	9	n/a	n/a	0.99
9-Item short form	716	9	0	9	9	n/a	n/a	0.99

**Figure 2:**
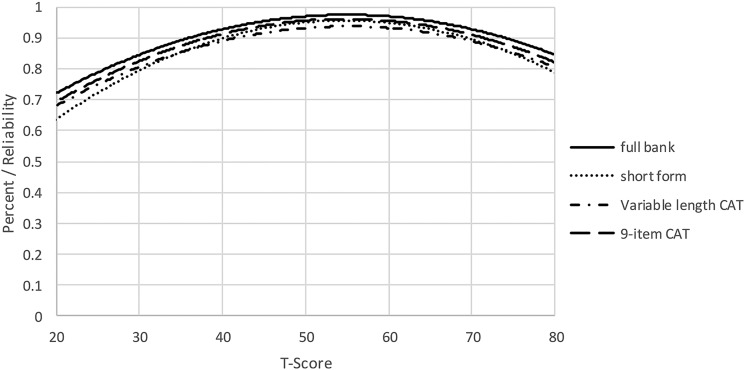
Measurement reliability by T-Score and assessment method.

To determine the degree of measurement precision and error for these assessments, we compared the reliability of the full bank, 9-item short form, and variable-length CAT with the default minimum of 4 items. When we compared the reliability of a CAT that was either fixed to 8 items, or a variable-length CAT with a minimum of 8 items, CAT values for both reliability (Fig. 2) and precision (Table 4) demonstrated improvement over the short form values. Table 4 presents the mean, standard deviation, range, and standard error ranges for the various administration modes. Additionally, reliability curves for the full bank, short form, variable length CAT (minimum of 4 items) and fixed-length CAT (8 items) are given in Fig. 2.

**Table 4:** Breadth of coverage for variable length CAT, fixed length CAT, 9-item short form, and full item bank

Mode	*N*	T score				Standard error	
		Mean ± SD	Range	% Ceiling	% Floor	Mean ± SD	Range
Variable-length CAT (min 4)	716	50.0 ± 9.6	29.4–79.8	0.3%	4.9%	0.30 ± 0.05	0.26–0.49
Variable-length CAT (min 8)	716	50.0 ± 9.6	29.4–79.8	0.3%	4.9%	0.56 ± 0.06	0.20–0.49
9-Item fixed-length CAT	716	49.9 ± 9.6	29.9–79.1	0.3%	5.4%	0.25 ± 0.07	0.19–0.49
9-Item short form	716	50.0 ± 9.5	31.0–76.0	1.4%	6.2%	0.27 ± 0.08	0.20–0.51
Full bank	716	49.9 ± 9.7	29.1–80.0	0.3%	4.8%	2.18 ± 0.75	0.16–0.48

### Scoring

SCI-QOL Grief and Loss scores are standardized on a T-metric, with a mean of 50 and a standard deviation of 10; this is based on the SCI-QOL calibration data; that is, a mean of 50 reflects the mean of an SCI population rather than the general population. All CAT administrations of the SCI-QOL Grief and Loss item bank are automatically scored by Assessment Center. When administering the short form, whether via Assessment Center, paper and pencil, or another data capture platform, an individual must complete all 8 component items in order to receive a score. The raw score for the short form is computed by simply summing the response scores for the individual component items. The T-score and associated standard error for each raw score value is given in Table 5.

**Table 5:** T-score lookup table for SCI-QOL Grief/Loss SF9a

Raw score	Scaled score	Standard error
9	30.9	5.1
10	35.3	3.9
11	37.4	3.6
12	39.4	3.3
13	41.0	3.1
14	42.3	2.9
15	43.6	2.8
16	44.7	2.7
17	45.8	2.6
18	46.7	2.5
19	47.7	2.5
20	48.6	2.5
21	49.4	2.4
22	50.3	2.4
23	51.1	2.4
24	51.9	2.4
25	52.7	2.4
26	53.5	2.4
27	54.3	2.4
28	55.1	2.4
29	55.9	2.4
30	56.7	2.4
31	57.5	2.4
32	58.3	2.4
33	59.1	2.4
34	60.0	2.4
35	60.8	2.5
36	61.8	2.5
37	62.7	2.5
38	63.7	2.6
39	64.7	2.7
40	65.9	2.8
41	67.2	2.9
42	68.6	3.0
43	70.2	3.3
44	72.3	3.5
45	76.1	4.4

### Reliability

As a part of the reliability study described in the Tulsky *et al.*^25^ methods paper in this issue, we compared Grief/Loss scores at Baseline with those from the 1-2 week retest assessment. In a sample of 245 individuals with SCI, Pearson's *r* = 0.84 and ICC (2,1) = 0.83 (95% CI = 0.78 to 0.87).

## Discussion

We developed the SCI-QOL Grief and Loss item bank to assess an individual's subjective experience of losses that is relevant to people with SCI. This new item bank is a departure from the status quo of currently available grief measures that focus primarily on death of a loved one. In contrast, items in the new item bank reflect losses of a life before injury, vision of a future life, time and opportunities, and the self they used to be. All of the items in the Grief and Loss item bank were developed directly from verbatim interview or focus group quotes; no items were drawn from PROMIS or Neuro-QOL. As a result, the new SCI-QOL Grief and Loss item bank fills an important gap in existing grief and bereavement instruments as well as these important patient oriented outcome measurement initiatives.

The use of IRT to calibrate the SCI-QOL Grief and Loss items has yielded a variety of administration options including a short form and CAT. The CAT outperformed the short form in reliability and precisions; as such, if a user's goal is to optimize reliability, especially at the ceiling and floor of the distribution, we would recommend administering the Grief and Loss item bank as a CAT with a minimum of 8 items. In cases where it may not be feasible or practical to administer items via CAT/Assessment Center, or if having participants answer the same subset of 8 items is necessary to answer a given research question, we would recommend short form administration. An additional administration option is to administer both the CAT and any short form items not included in the CAT by using the ‘no duplicates’ option in Assessment Center. In this way, the user could optimize reliability and have the option of directly comparing individuals' responses on specific items to each other or to themselves over time.

### Clinical applications

The flexibility of methods to administer the SCIQOL Grief and Loss item bank provides scientists and clinicians with an efficient and accessible way to integrate the measurement of grief and loss specific to SCI into research and ultimately clinical practice. Next, research is needed to examine the validity of this Grief and Loss measure. For example, studies are needed to determine how grief symptoms change over time and whether grief and loss can be discriminated from depression and anxiety in people with SCI. It will also be important to determine whether the magnitude or persistence of grief and loss predict other important outcomes such as quality of life, functional impairment, and self-care after controlling for depression, anxiety and other salient variables. If, as is the case with other type of loss,^14^ there is a significant minority of persons who have prolonged, disabling grief symptoms, tailored grief treatment approaches should be studied.^16^

The integration of this measure in clinical practice can be particularly useful for guiding therapy to target specific areas of difficulty. For example, losses of a future, loss of a sense of self, or acceptance of injury all have important implications for tailoring therapy to individual needs. Use of the measure in clinical settings can also aid the clinician for tracking change over time to assess the effectiveness of therapy and guide changes in their approach. By design, interpretation of SCI-QOL scores is simplified by a standardized metric that allows the clinician to readily identify how the individual compares to the broader SCI population. Finally, the use of this new measure can aid clinicians in the distinguishing grief and loss from depression to guide treatment.

## Conclusion

The final SCI-QOL Grief and Loss item bank contains 17, IRT-calibrated items. This item bank is the first, to our knowledge, to specifically capture grief and loss after SCI. Because of the flexibility of IRT-based measures, the use of CATs is also possible with this item bank, enabling researchers and clinicians to administer only the most precise and informative items based on an individual's responses. This has important implications for the use of such innovative applications in symptom monitoring and self-management in post-acute care settings and community living.

To the best of our knowledge, this is the first time that a patient-centered, modern measurement theory derived approach has been used to develop and test a grief and loss self-reported measurement tool specifically for individuals with SCI. Our work using focus groups and interviews strengthened our understanding of grief and loss as it is experienced in the context of SCI. While this work has made great strides forward, there remains work to be done to more comprehensively define grief and loss in this population. This new item bank makes an important first step towards reliably measuring the construct of grief and loss in persons with SCI. Such measurement will significantly contribute to the development of conceptual models. Future work will also include establishing discriminant validity from depression and other grief measures to further ensure that the SCI-QOL Grief and Loss item bank captures the unique losses experienced after injury. The knowledge gained from the use of this new measure will be essential for the development of grief and loss conceptual models to inform treatment approaches to maximize HRQOL after SCI.

## Disclaimer statements

**Contributors** All authors have contributed significantly to the design, analysis and writing of this manuscript. The contents represent original work and have not been published elsewhere.

**Funding** This study was supported by grant #5R01HD054659 from the National Institutes of Health – Eunice Kennedy Shriver National Institute of Child's Health and Human Development/National Center on Medical Rehabilitation Research and the National Institute on Neurological Disorders and Stroke.

**Conflicts of interest** No commercial party having a direct financial interest in the results of the research supporting this article has or will confer a benefit upon the authors or upon any organization with which the authors are associated. All SCI-QOL items and parameters are © 2015 David Tulsky and Kessler Foundation. All rights reserved. All items are freely available to the public via the Assessment Center platform (http://www.assessmentcenter.net). There are currently no plans for Dr. Tulsky or Kessler Foundation to benefit financially from the copyright.

**Ethics approval** The Institutional Review Board at each site reviewed and approved this project.
